# The Toxicity of Nanoparticles Depends on Multiple Molecular and Physicochemical Mechanisms

**DOI:** 10.3390/ijms18122702

**Published:** 2017-12-13

**Authors:** Yue-Wern Huang, Melissa Cambre, Han-Jung Lee

**Affiliations:** 1 Department of Biological Sciences, Missouri University of Science and Technology, Rolla, 143 Schrenk Hall, 1870 Miner Circle, Rolla, MO 65409, USA; mhcxv8@mst.edu; 2 Department of Natural Resources and Environmental Studies, National Dong Hwa University, Hualien 97401, Taiwan; hjlee@mail.ndhu.edu.tw

**Keywords:** nanoparticle, toxicity, physicochemical property, cell proliferation, calcium homeostasis, oxidative stress

## Abstract

Nanotechnology is an emerging discipline that studies matters at the nanoscale level. Eventually, the goal is to manipulate matters at the atomic level to serve mankind. One growing area in nanotechnology is biomedical applications, which involve disease management and the discovery of basic biological principles. In this review, we discuss characteristics of nanomaterials, with an emphasis on transition metal oxide nanoparticles that influence cytotoxicity. Identification of those properties may lead to the design of more efficient and safer nanosized products for various industrial purposes and provide guidance for assessment of human and environmental health risk. We then investigate biochemical and molecular mechanisms of cytotoxicity that include oxidative stress-induced cellular events and alteration of the pathways pertaining to intracellular calcium homeostasis. All the stresses lead to cell injuries and death. Furthermore, as exposure to nanoparticles results in deregulation of the cell cycle (i.e., interfering with cell proliferation), the change in cell number is a function of cell killing and the suppression of cell proliferation. Collectively, the review article provides insights into the complexity of nanotoxicology.

## 1. Introduction

Nanoscience is the study of the control of matters at the atomic and molecular scale. Nanomaterials are materials that have at least one dimension in the range of 1–100 nm. In addition to discovering fundamental principles and advancing knowledge in nanoscience, nanomaterials have a wide spectrum of applications in our society. Table 1 summarizes the industrial applications of transition metal oxide nanoparticles [1,2,3,4,5,6,7,8,9,10,11,12,13,14,15,16,17,18,19,20,21,22,23,24]. Some engineered nanomaterials are being used in products with direct exposure to humans. For example, TiO_2_ nanoparticles are used in food coloring, cosmetics, skin care products, and tattoo pigment [1,2,3,4,5,6,7]. Fe_2_O_3_ nanoparticles are used in the final polish on metallic jewelry. ZnO nanoparticles are added to many products including cotton fabric, food packaging, and rubber for its deodorizing and antibacterial properties [18,19,20]. Engineered nanomaterials also show promise for applications in life science and biomedical utility such as cellular receptor trafficking, delivery of biologically active molecules, disease staging and therapeutic planning, and nanoelectronic biosensors [25,26]. For instance, nanoparticles incorporated with targeting ligands can enter cancer cells, where they can release therapeutic drugs [25]. This could decrease the amount of drug needed to treat a disease (i.e., higher therapeutic efficacy) as well as unwanted side effects (toxicity). There are more than 3000 nanoparticulate-based commercial applications. By the end of 2019, its worldwide market is estimated to be $79.8 billion [27]. As the use of engineered nanomaterials continues to grow exponentially, unintended and intended exposure may occur, leading to a greater degree of human health risk. The exposure routes may include inhalation, ingestion, skin, and injection. End-product users, occupational exposed subjects, and the general public may be at risk of adverse effects. The use of nanomaterials has significantly grown in the automotive, construction, enerty, biomedical, electronic, textile, chemical, and cosmetic industries [28]. Uncovering the specific particle surface properties that cause some to be more toxic than others requires a systematic study focusing on nanoparticles similar in composition (size and morphology). Therefore, we choose to focus on transition metal oxide nanoparticles widely used in various industrial applications.

## 2. Characteristics of Nanoparticles that Influence Toxicity

The physiochemical properties of nanoparticles influence how they interact with cells and, thus, their overall potential toxicity. Understanding these properties can lead to the development of safer nanoparticles. Recent studies have begun identifying various properties that make some nanoparticles more toxic than others. Theoretically, particle size is likely to contribute to cytotoxicity. Given the same mass, smaller nanoparticles have a larger specific surface area (SSA) and thus more available surface area to interact with cellular components such as nucleic acids, proteins, fatty acids, and carbohydrates. The smaller size also likely makes it possible to enter the cell, causing cellular damage. In some nanoparticles, toxicity was found to be a function of both size and SSA. For instance, the size of anatase TiO_2_ was shown to correlate with reactive oxygen species (ROS) production when comparing the amount of ROS production per surface area within a certain size range [29]. Particles below 10 or above 30 nm produced similar levels of ROS per surface area. However, there was a dramatic increase in ROS production per unit surface area in particles increasing from 10 to 30 nm. This information provides insight regarding the complex relationship between nanoparticle properties and nanotoxicity. Further studies are needed to determine whether a similar phenomenon applies to other forms of TiO_2_ or other particles.

Particle surface charge may affect the cellular uptake of particles as well as how the particles interact with organelles and biomolecules. Consequently, particle surface charge influences cytotoxicity. According to mathematical probability and assuming particles are toxic, high particle uptake (i.e., higher bioavailability) correlates with higher toxicity. For instance, three similarly sized iron oxide particles with different charges were found to have differential toxicities on a human hepatoma cell line (BEL-7402) [30]. Oleic acid-coated Fe_3_O_4_, carbon-coated Fe, and Fe_3_O_4_ had surface charges of 4.5, 23.7, and 14.5 mV, respectively. The toxicity of the nanoparticles increased with an increase in surface charge. This suggests that the higher positive charge the nanoparticle has, the greater electrostatic interactions it has with the cell and, thus, greater endocytic uptake. Another example is that positively charged ZnO nanoparticles produce more cytotoxic effects in A549 cells than negatively charged particles of a similar shape and size [31]. The phenomenon can be explained, in part, in the context of cellular membrane composition. Glycosaminoglycans are abundant on the mammalian cell surface. These molecules are negatively charged and therefore are likely to interact electrostatically with positively charged nanoparticles [32]. The longer and the more the electrostatic interactions, the more likely nanoparticles are to be internalized [33]. The same is true in positively charged nanoparticles interacting with negatively charged DNA, leading to DNA damage.

Shape also affects levels of toxicity. Amorphous TiO_2_ was found to generate more ROS than anatase or rutile of a similar size, with rutile TiO_2_ causing the least amount of ROS [29]. It is likely that amorphous TiO_2_ has more surface defects, and therefore active sites that are capable of causing ROS. The anatase form of TiO was also significantly more toxic to PC12 cells than the rutile form even though the particles are similar in size and chemical make-up [34]. Rod-shaped Fe_2_O_3_ nanoparticles were found to produce much higher cytotoxic responses than sphere-shaped Fe_2_O_3_ nanoparticles in a murine macrophage cell line (RAW 264.7), including higher levels of lactate dehydrogenase (LDH) leakage, inflammatory response, ROS production, and necrosis [35]. Finally, rod-shaped CeO_2_ nanoparticles were found to produce more toxic effects in RAW 264.7 cells than octahedron or cubic particles [36]. Rod-shaped CeO_2_ nanoparticles produced significant lactate dehydrogenase LDH release and tumor necrosis factor alpha (TNF) in RAW 264.7 cells, while neither octahedron nor cubic produced significant responses. Why the physical shape of a nanoparticle influences cytotoxicity remains to be elucidated.

**Figure 1:**
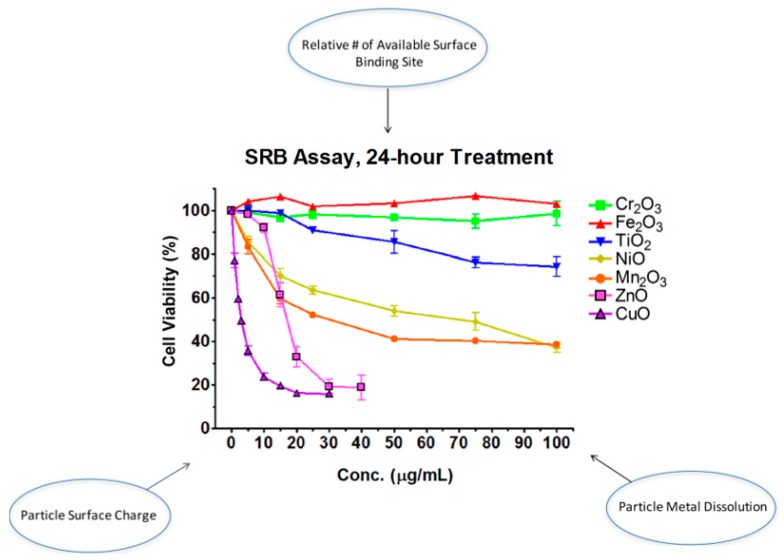
Certain physicochemical parameters of transition metal oxide nanomaterials influence toxicity.

Though the above studies and others have contributed to the understanding of how and why properties of nanoparticles mediate toxicity, a more systematic approach can even further advance our knowledge in this regard. Our laboratory systematically selected seven oxides of transition metals (Ti, Cr, Mn, Fe, Ni, Cu, and Zn) from the fourth period of the periodic table of elements [33]. Four properties of nanomaterials were tested: particle surface charge, available binding site on particle surface, particle metal dissolution, and band-gap energy (Figure 1). Particle surface charge was determined by point-of-zero charge (PZC). We used X-ray photoelectron spectroscopy (XPS) to measure available binding site on particle surface. Metal ions released from oxides were analyzed with inductively coupled plasma mass spectrometry (ICP-MS). Finally, bad-gap energy, which is the energy difference between the top of the valence band and the bottom of the conduction band in insulators and semiconductors, was spectroscopically determined. We found that (1) as the atomic number of the element increases, cytotoxicity increases; and (2) alteration of cell viability is a function of particle surface charge, available binding site on a particle surface, and particle metal dissolution, but not of band-gap energy.

## 3. Biochemical and Molecular Mechanisms of Cytotoxicity

**Figure 2:**
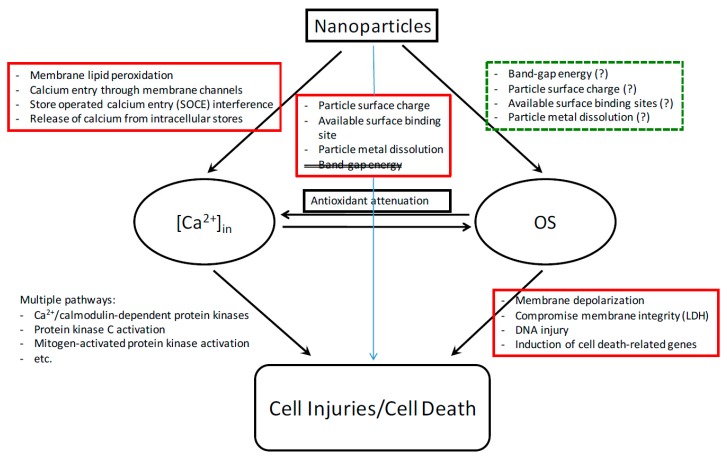
Multiple mechanisms of nanoparticle toxicity contribute to cell cycle deregulation and cell death. Particles used to delineate the pathways include Al_2_O_3_, SiO_2_, CeO_2_, and transition metal oxides.

There have been intensive nanotoxicological studies since the turn of the century [37,38,39,40]. Mechanisms of in vivo nanotoxicity are numerous. They may include, but not limited to, pulmonary and systemic inflammation, platelet activation, altered heart rate variability, and vasomotor dysfunction [41]. While in vivo studies provide critical information for risk assessment, in vitro studies help us understand molecular and biochemical mechanisms of nanotoxicity and give insight into the physicochemical properties of nanomaterials that contribute to the toxicity. For instance, metal oxide nanoparticles can elevate the level of oxidative stress (OS) via production of reactive oxygen species (ROS; e.g., O_2_^•−^, OH^•^, H_2_O_2_) in a variety of ways [42]. These high-energy species can attack lipids, nucleic acids, proteins, and other essential biomolecules. The consequential damage includes damage to mitochondrial structure, depolarization of mitochondrial membrane, impairment of the electron transport chain, and the activation of an NADPH-like system [43]. Our laboratory has focused on delineating multiple biochemical and molecular mechanisms of toxicity induced by exposure to a variety of nanoparticles (Figure 2). The nanoparticles tested can elevate cellular OS, which is manifested in reduced levels of the antioxidants GSH and α-tocopherol [44,45]. This leads to cellular injury or death via altered signaling pathways. Compromise of cell membrane integrity is detected via release of LDH from the cell [44,45]. DNA injuries, including double-strand and single-strand breakages, are identified according to the comet assay [46]. DNA damage can lead to cell cycle arrest or apoptosis. An oxidative stress and antioxidant defense microarray assay found alterations in the expression of four genes that are involved in apoptosis and OS responses: BNIP, PRDX3, PRNP, and TXRND1 [47]. Membrane depolarization occurs in cells treated with aluminum oxide (AL_2_O_3_) and cerium oxide (CeO_2_) [48].

In addition to OS, we observed nanoparticle-induced perturbation of intracellular calcium [Ca^2+^] in homeostasis, which can be attributed to several molecular actions and is associated with metabolic and energetic imbalance as well as cellular dysfunction [47] (Figure 2). Zinc oxide (ZnO) nanoparticles increase [Ca^2+^]_in_. The moderation of this increase by nifedipine suggests that a portion of this increase reflects an influx of extracellular calcium. Membrane disruption (e.g., by the demonstrated lipid peroxidation, malondialdehyde MDA) may also play a role in this influx. Nanomaterials disrupt store-operated calcium entry [49,50]. There exist crosstalks between intracellular [Ca^2+^]_in_ and OS, and the increases in both can be reduced by an antioxidant. Finally, while [Ca^2+^]_in_ and OS affect the activity of each other, they induce cell death by distinct pathways. These findings suggest that nanomaterials can trigger cell death via multiple pathways.

Studies have shown a decrease in mitochondrial membrane potential (MMP) upon exposure to ZnO in human bronchial epithelial cells (BEAS-2B) and human alveolar adenocarcinoma cells (A549) as detected by the MitoTracker^®^ Red CMXRos and JC-1 assay, which indicate risk of early apoptosis [51]. TiO_2_ causes a loss of MMP in neuronal cells (PC12) and lung A549 cells [34,52]. Fe_3_O_4_ caused a loss of MMP in human mesenchymal stem cells (hMSCs) [53] and human hepatoma cells (BEL-7402) [30]. TEM images show that ZnO nanoparticles appeared to physically squeeze mitochondrial cells in HaCaT cells, likely one mechanism of mitochondrial damage [54]. Recent studies investigated protein deregulation by metal oxide nanoparticles [55]. Using circular dichroism (CD), Fourier transformed infrared spectrometry (FTIR), fluorescence spectroscopy (FS), Raman spectroscopy (RS), and nuclear magnetic resonance (NMR), the binding of proteins to ZnO, TiO_2_, SiO_2_, or FeO nanoparticles can result in minor conformational changes or protein denaturation, an irreversible binding of proteins to a nanoparticle [55]. Furthermore, metal ions such as Zn^2+^ and Cu^2+^ released from ZnO and CuO can cause damage to proteins. Metal ions such as copper and zinc can inactivate certain metalloproteins by dislodging metal ions within them [56]. Another mechanism of nanotoxicity pertains to cell cycle arrest. Deregulation of cell cycle occurs in cells exposed to TiO_2,_ Fe_2_O_3,_ CuO, NiO, ZnO, and Al_2_O_3_ [30,34,51,52,53,54,57,58,59,60,61,62,63,64,65,66,67,68] (Table 2). Cells in cell cycle arrest will either exit cell cycle arrest with potentially compromised cellular function or undergo apoptosis.

## 4. Mechanisms of Cell Cycle Arrest

While previous studies have been focusing on alteration of cell viability, recent studies have demonstrated that a change in cell number in cytotoxicity tests reflects not just cell killing but also cell cycle arrest, which leads to a suppression of cell proliferation. Therefore, studies on cell cycle arrest aid a better understanding of the reduction of viable cells. The suppression of cell proliferation occurs when cells become arrested in one or more cell cycle phases. Cell growth can become arrested in the G_0_/G_1_ phase, the S phase, or the G_2_/M phase. The phase in which cell growth becomes arrested is cell-type- and nanoparticle-specific [30,34,51,52,53,54,57,58,59,60,61,62,63,64,65,66,67,68]. Table 2 demonstrates various changes in cell cycle upon exposure to different nanoparticles in a variety of cell lines. Certain nanoparticles are likely to cause DNA damage, which may lead to cell cycle arrest. Cells arrested in cell cycle will either fix the damage or accumulate too much damage and undergo apoptosis. While the underlying mechanisms in which cells become arrested in certain phases of the cell cycle vary, all cells undergoing cell cycle arrest experience a suppression of proliferation. The degree to which cells experience an inhibition of proliferation influences cell number from one generation to the next.

### 4.1. Cell-Type-Dependent Suppression of the Cell Cycle

Exposure of nickel oxide nanoparticle (NiONP) resulted in a significant increase in the G_0_/G_1_ in the BEAS-2B cell line but a significant decrease of the G_0_/G_1_ phase in the A549 cell line [57] Consequently, exposure to NiONP resulted in a significant decrease in the G_2_/M in the BEAS-2B cell line and a significant increase of the G_2_/M phase in the A549 cell line. However, the S phase was only significantly affected in the BEAS-2B cell line. Furthermore, exposure to ZnO caused an increase in the population of cells in the G_2_/M phase in A549 cells but did not affect cell cycle distribution in BEAS-2B cells. [51]. These studies demonstrate that cell cycle arrest is cell-type-specific, evidence of cellular stress activating different response pathways in different cell types.

### 4.2. Nanoparticle Dependent Suppression of Cell Cycle

Cell cycle arrest also differs based on the type of nanoparticle. It appears that cell cycle arrest occurs most commonly in the G_2_/M phase. However, arrest can also happen in the G_0_/G_1_ and S phases. In BEAS-2B cells, exposure to NiO caused cells to become arrested in the G_0_/G_1_ phase, while exposure to ZnO and Fe_2_O_3_ did not affect the cell cycle [51,57]. ZnO and CuO exposure resulted in arrest in the G_2_/M phase, while TiO_2_ exposure resulted in arrest in the S phase in HaCaT cells [54,58,62]. Al_2_O_3_ and Fe_3_O_4_ caused an increase in the sub-G_0_ phase of human mesenchymal stem cells (hMSFs) [53,63]. A549 cells became arrested in the G_2_/M phase upon exposure to CuO, NiO, and ZnO, but experience no change in cell cycle upon exposure to Fe_2_O_3_ [51,57,59,60]. One study found that TiO_2_ exposure caused A549 cells to become arrested in the G_0_/G_1_ phase, while two other studies found that exposure caused arrest in the G_2_/M phase [52,60,61]. This could be due to differences in TiO_2_’s size or other properties. Collectively, cell cycle alteration is a complex matter involving properties of both cells and particles.

### 4.3. Changes in Gene Expression Underlie the Mechanisms of Cell Cycle Arrest

Study of gene responses upon nanoparticle exposure can further enhance our understanding of the biological pathways in which nanoparticles induce cell cycle arrest. Cell cycle progression is regulated by a variety of growth factors that promote transition through various phases as well as inhibitors that prevent or decelerate transition. Exposure to nanoparticles can result in a wide array of gene expression deregulation pertaining to the cell cycle. For instance, exposure to CuO nanoparticles causes downregulation of 90 cell cycle genes [59]. Nanoparticle exposure can affect different genes in different cell lines upon exposure to the same nanoparticle. There is a cell-type-specific difference in the regulation of the cell cycle between a normal intestinal cell line NCM460 and two cancerous intestinal cell lines, DLD-1 and SW480 [69]. ZnO exposure induced the p53 pathway in NCM460 cells but not DLD-1 or SW480 cells. The mutated p53 function in the cancerous cell lines might have contributed to the observed difference. NCM460, DLD-1, and SW480 cell lines experienced an increase in checkpoint kinase 1 (Chk-1), leading to cell cycle arrest. Not all cancerous cell lines are incapable of inducing the p53 pathway. For instance, cancerous A549 cells experienced an increase in the expression of p53 upon exposure to TiO_2_ [61]. TiO_2_ was found to induce double-strand breaks and a downregulation of cyclin B1 (a protein involved in mitosis) in A549 cells, leading to cell cycle arrest in the G_2_/M phase [61]. CuO exposure causes the downregulation of various genes that allow cells to progress through the cycle at a couple of checkpoints in A549 cells [59]. Exposure of CuO downregulates proliferating cell nuclear antigen (PCNA, involved in proliferation), cell-division cycle protein (CDC2), and cyclin B1 (CCNB1, involved in G2 to M transition) [59]. ZnO exposure causes DNA damage and the downregulation of cyclin B1 and cyclin-dependent kinase 1 (CDK1) in human immortal keratinocyte cells (HaCaT), causing G2 arrest. PCNA was also downregulated [54]. Further studies are needed to demonstrate what genes cause cells to become arrested in the S or G_0_/G_1_ phase of the cell cycle. A systematic study looking at the gene responses after exposing a cell to different nanoparticles that lead to phase-specific changes in the cell cycle could provide evidence of how the characteristics of nanoparticles induce specific changes.

It is possible for cells in cell cycle arrest to recover and continue proliferating upon the removal of nanoparticles. A549 cells whose proliferation is halted by CuO exposure could start proliferating again if cultured in a fresh medium. Reduction of stress can also allow cells to recover from cell cycle arrest. For instance, ZnO nanoparticle exposure induces G_2_/M arrest in intestinal cell lines and the addition of antioxidant N-acetylcysteine can reverse cell cycle arrest by approximately 50–70% [69].

## 5. Cytotoxicity Is a Function of Cell Killing and Suppression of Proliferation

Numerous mechanisms may involve toxicity induced by exposure to nanoparticles. Altered signaling pathways perturb cellular homeostasis leading to cellular injuries. Nanotoxicity could lead to suppression of proliferation (via cell cycle arrest). When cells cannot overcome the stress and fix the damage, they are destined to death (apoptosis or necrosis). While the mechanisms that determine which cell cycle phase could become arrested are multiple, the consequential suppression of proliferation affects the cell number from one generation of cells to the next. Using the tritiated thymidine incorporation assay, we recently demonstrated that seven transition metal oxide nanoparticles can differentially suppress cell proliferation [70]. Assuming the doubling time of a cell line is 24 h and the rate of doubling time of cells is not altered, upon exposure to nanoparticles over a period of 24 h, the estimated number of cells in the second generation is expected to be as follows:

 Cell # in Generation 2=2(Proliferating cells)+non proliferating cells−dead cells

Future studies should weigh the contribution of these two independent variables to the alteration in cell number.

## 6. Conclusions

Nanotoxicology emerged approximately at the turn of the century. Numerous studies have been conducted to better understand the impact nanomaterials have on environmental and human health and help us move toward making safer materials. In vitro studies are essential to identify biochemical and molecular mechanisms of cytotoxicity as the complexities of toxicokinetics and toxicodynamics typically observed in animal studies do not exist. In vitro studies provide insight to hazard identification which can lead to further studies on animal subjects. They are also the first step in identifying occupational risk assessment. Cumulative studies could potentially lead to a characterization model that allows workers to become aware of the potential risks of nanoparticle exposure. Preliminary data from in vitro experiments can potentially provide a precautionary risk management system in which workers are educated on the nanoparticles that have been shown to produce toxic and carcinogenic effects in in vitro experiments [28]. Properties of nanoparticles that contribute to cytotoxicity include, but are not limited to, surface, particle size, particle morphology, and dissolution of ions. As oxidative stress is elevated and intracellular calcium homeostasis is perturbed due to exposure to nanoparticles, subsequent actions lead to cell injury and death, and deregulation of the cell cycle. The change in cell number is a function of cell killing and the suppression of proliferation. Deregulation of the cell cycle could result in cell death, non-proliferation, or recovery (upon removal of nanoparticles). Although the scientific community has made considerable strides in understanding nanotoxicity in the recent past, the future research needed to decipher nanotoxicity remain significant. For instance, what are the properties of the nanoparticle that induce oxidative stress? How do nanoparticles interact, physically and chemically, with biomolecules such as nucleic acids, proteins, and lipids leading to alteration of gene expression? What is the basic scientific principle that dictates the shape-dependent cytotoxicity? Last but not least, quantification of cellular uptake of nanoparticles using single-particle ICP-MS may help with (1) the correlation of dose–effect and (2) the contribution of dissolved ions to cytotoxicity. As more information is gathered, it may be possible to apply the concept of quantitative structure and activity relationship (QSAR) to systematically delineate the cause–effect relationship. This could further improve the safety of the nanomaterial worker.

## Figures and Tables

**Table 1:** Applications of transition metal oxide nanoparticles.

Elements	Oxide	Potential Application
Scandium (Sc)	Sc_2_O_3_	Used in high-temperature systems for its resistance to heat and thermal shock, electronic ceramics, and glass composition
Titanium (Ti) [1,2,3,4,5,6,7]	TiO_2_	White pigment, white food coloring, cosmetic and skin care products, thickener, tattoo pigment and styptic pencils, plastics, semiconductor, solar energy conversion, solar cells, solid electrolytes, detoxification or remediation of wastewater; used in resistance-type lambda probes; can be used to cleave protein that contains the amino acid proline at the site where proline is present, and as a material in the meristor
Vanadium (V)	V_2_O_5_	Catalyst, a detector material in bolometers and microbolometer arrays for thermal imaging, and in the manufacture of sulfuric acid, vanadium redox batteries; preparation of bismuth vanadate ceramics for use in solid oxide fuel cells [8]
	V_2_O_3_	Corundum structure as an abrasive [9], antiferromagnetic with a critical temperature at 160 K [10] can change in conductivity from metallic to insulating
Chromium (Cr)	Cr_2_O_3_	Protection of silicon surface morphology during deep ion coupled plasma etching of silica layers; used in paints, inks, and is the precursor to the magnetic pigment chromium dioxide
	CrO_2_	Magnetic tape emulsion, data tape applications
Manganese (Mn)	MnO_2_	Electrochemical capacitor, as a catalyst; used in industrial water treatment plants
Iron (Fe)	Fe_2_O_3_	Used as contrast agents in magnetic resonance imaging, in labeling of cancerous tissues, magnetically controlled transport of pharmaceuticals, localized thermotherapy, preparation of ferrofluids [11,12], final polish on metallic jewelry and lenses, as a cosmetic
	FeO	Tattoo inks
	Fe_3_O_4_	MRI scanning [13], as a catalyst in the Haber process and in the water gas shift reaction [14], and as a black pigment [15]
Cobalt (Co)	Co_2_O_3_	Catalyst; for studying the redox and electron transfer properties of biomolecules; can immobilize protein
	CoO	Blue colored glazes and enamels, producing cobalt(II) salts
Nickel (Ni)	NiO	In ceramic structures, materials for temperature or gas sensors, nanowires and nanofibers, active optical filters, counter electrodes
	Ni_2_O_3_	Electrolyte in nickel plating solutions; an oxygen donor in auto emission catalysts; forms nickel molybdate, anodizing aluminum, conductive nickel zinc ferrites; in glass frit for porcelain enamel; thermistors, varistors, cermets, and resistance heating element
Copper (Cu)	CuO	Burning rate catalyst, superconducting materials, thermoelectric materials, catalysts, sensing materials, glass, ceramics, ceramic resisters, magnetic storage media, gas sensors, near infrared tilters, photoconductive applications, photothermal applications, semiconductors, solar energy transformation [16]; can be used to safely dispose of hazardous materials [17]
	Cu_2_O	Pigment, fungicide, antifouling agent for marine paints, semiconductor
Zinc (Zn)	ZnO	Added to cotton fabric, rubber, food packaging [18,19,20], cigarettes [21], field emitters [22], nanorod sensors; Applications in laser diodes and light emitting diodes (LEDs), a biomimic membrane to immobilize and modify biomolecules [23]; increased mechanical stress of textile fibers [24]

**Table 2:** Changes in cell cycle upon exposure to nanoparticles with a variety of characteristics in various cell lines.

Cell Line	Nanoparticle	Size (nm)	Specific Surface Area (m^2^/g)	Zeta Potential (mV)	Shape	Effect on Cell Cycle	Reference
Human alveolar adenocarcinoma (A549)	TiO_2_	>100	---	---	irregular	↑G_0_/G_1_	[59]
A549	Fe_2_O_3_	39.2 *	---	---	spherical	No change	[50]
A549	CuO	50	---	−23.96 **	sphere	↑G_2_/M	[58,66]
A549	CuO	>50	---	---	irregular	↑G_2_/M	[59]
A549	NiO	50, 80 *, 450 **	61.16	−12; −22	---	↑G_0_/G_1_	[56]
						↑G_2_/M	
						↑sub G_0_	
A549	ZnO	63.1 *	---	---	nearly spherical	↑G_2_/M	[50]
A459	TiO_2_	23.28 ± 2.0 **	12–15	−10.16 ± 1.0 **	anatase	↑G_2_/M	[60]
		106.7 ± 8.0 *					
		4–8		−13 ± 0.9 *			
A549	TiO_2_	<5	200	−0.55 **	anatase	↑G_2_/M	[51]
		65.3 **				↓G_0_/G_1_	
Human bronchial epithelial cells (BEAS-2B)	Fe_2_O_3_	39.2 *	---	---	spherical	No change	[50]
BEAS-2B	NiO	50	---	−12/−22	---	↑G_0/_G_1_	[56]
						↓G_2_/M	
						↓S	
						↑Sub G_0_	
BEAS-2B	ZnO	63.1 *	---	---	nearly spherical	No change	[50]
Human immortal keratinocyte cells (HaCaT)	TiO_2_	12 **	---	−11.9 ± 0.8 **	spherical	↓G_0_/G_1_	[57]
						↑S	
HaCaT	ZnO	<100	15–25	−12.6 ± 0.95 **	rod-shaped	↑G_2_/M	[53]
		132.55 ± 0.45 **				↓S	
HaCaT	CuO	3–6 *	---	~37.5 *	---	↑G_2_/M	[61]
						↓G_0_/G_1_	
						↓S	
Rat pheochromocytoma (PC12)	TiO_2_	20	---	−12.5	anatase	↑G_2_/M	[33]
Rat PC12	TiO_2_	20	---	−23.2	rutile	↑G_2_/M	[33]
Human neuroplastoma (SHSY5Y)	ZnO	100	15–20	−8.23 *	---	↓G_0/_G_1_	[65]
		243.7 *		−11.7 **		↓G_2_/M	
		273.4 **				↑S	
Human mesenchymal stem cells (hMSFs)	Al_2_O_3_	20–100	---	---	spherical	↓G_0/_G_1_	[62]
						↓G_2/_M	
		205 *				↑Sub G_0_	
Human hMSFs	Fe_3_O_4_	50–75	---	---	spherical	↓G_0_/G_1_	[52]
		119 *				↑Sub G_0_	
		210 **					
Human hepatoma (BEL-7402)	Fe_3_O_4_	10–30	---	14.4	---	↑G_0/_G_1_	[29]
						↓S	
Human epidermal carcinoma (A431)	ZnO	215.8 ± 0.1 *	---	−25.3 ± 0.4 *	---	↑S	[64]
		30.9 ± 0.5 *		−12.8 ± 0.6 **		↑G_2_/M	
*Allium cepa* root cells	ZnO	75–85	---	---	mostly cuboidal to hexagonal-octagonal, some rod	↓G_0/_G_1_	[63]
						↑G_2_/M	
						↑Sub G_0_	
Mouse embryonic fibroblast (MEF)	CuO	3–6	---	~37.5	---	↑G_2_/M	[61]
						↓G_0_/G_1_	
						↓S	
*Xenopus laevis* (A6)	Poly-CuO	100	---	---	---	↑G_2_/M	[67]
		40–500 *				↓S	
*Xenopus laevis* (A6)	CuO	6 ± 1	---	---	---	↑G_2_/M	[67]
		9–40 *					

* Measured in water, ** Measured in cell culture medium, ↓ Decrease in cell number, ↑ Increase in cell number, --- Data not available.

## References

[1] Jones B.J., Vergne M.J., Bunk D.M., Locascio L.E., Hayes M.A. (2007). Cleavage of Peptides and Proteins Using Light-Generated Radicals from Titanium Dioxide. Anal. Chem..

[2] TIME TIME’s Best Inventions of 2008. http://content.time.com/time/specials/packages/article/0,28804,1852747_1854195_1854176,00.html.

[3] Earle M.D. (1942). The Electrical Conductivity of Titanium Dioxide. Phys. Rev..

[4] Hogan J. (2004). Smog-busting paint soaks up noxious gases. New Scientist.

[5] Phillips L.G., Barbano D.M. (1997). The Influence of Fat Substitutes Based on Protein and Titanium Dioxide on the Sensory Properties of Lowfat Milks. J. Dairy Sci..

[6] Fujishima A. (2005). Discovery and applications of photocatalysis—Creating a comfortable future by making use of light energy. Jpn. Nanonet Bull..

[7] Fujishmia A., Honda K. (1972). Electrochemical Photolysis of Water at a Semiconductor Electrode. Nature.

[8] Vaidhyanathan B., Balaji K., Rao K.J. (1998). Microwave-Assisted Solid-State Synthesis of Oxide Ion Conducting Stabilized Bismuth Vanadate Phases. Chem. Mater..

[9] Greenwood N.N., Earnshaw A. (1997). Chemistry of the Elements.

[10] Page E.M., Wass S.A. (1994). Vanadium:Inorganic and Coordination chemistry. Encyclopedia of Inorganic Chemistry.

[11] Adlam G.H.J., Price L.S. (1945). Higher School Certificate Inorganic Chemistry.

[12] Greedon J.E., King R.B. (1994). Magnetic oxides. Encyclopedia of Inorganic Chemistry.

[13] Babes L., Denizot B., Tanguy G., Jacques Le Jeunne J., Jallet P. (1999). Synthesis of Iron Oxide Nanoparticles Used as MRI Contrast Agents: A Parametric Study. J. Colloid Interface Sci..

[14] Lee S. (2005). Encyclopedia of Chemical Processing.

[15] Buxbaum G., Pfaff G. (2005). Industrial Inorganic Pigments.

[16] AZoNano Copper Oxide (CuO) Nanoparticles—Properties, Applications. https://www.azonano.com/article.aspx?ArticleID=3395.

[17] Kenney C.W., Uchida L.A. Use of Copper (II) Oxide as Source of Oxygen for Oxidation Reactions. http://www.freepatentsonline.com/4582613.html.

[18] Saito M. (1993). Antibacterial, Deodorizing, and UV Absorbing Materials Obtained with Zinc Oxide (ZnO) Coated Fabrics. J. Ind. Text..

[19] Li Q., Chen S.-L., Jiang W.-C. (2007). Durability of nano ZnO antibacterial cotton fabric to sweat. J. Appl. Polym. Sci..

[20] Akhavan O., Ghaderi E. (2009). Enhancement of antibacterial properties of Ag nanorods by electric field. Sci. Technol. Adv. Mater..

[21] AZoNano Zinc Oxide (ZnO) Nanoparticles—Properties, Applications. https://www.azonano.com/article.aspx?ArticleID=3348.

[22] Li Y.B., Bando Y., Golberg D. (2004). ZnO nanoneedles with tip surface perturbations: Excellent field emitters. Appl. Phys. Lett..

[23] Kumar S.A., Chen S.M. (2008). Nanostructured Zinc Oxide Particles in Chemically Modified Electrodes for Biosensor Applications. Anal. Lett..

[24] Qin Y., Wang X., Lin Wang Z. (2008). Editor’s summary: Nanomaterial: Power dresser. Nature.

[25] Choi C.H., Alabi C.A., Webster P., Davis M.E. (2010). Mechanism of active targeting in solid tumors with transferrin-containing gold nanoparticles. Proc. Natl. Acad. Sci. USA.

[26] Korin N., Kanapathipillai M., Ingber D.E. (2012). Sheer-responsive platemet mimetics for targeted drug delivery. Isr. J. Chem..

[27] Highsmith J. (2014). Nanoparticles in Biotechnology, Drug Development and Drug Delivery. Global Markets: A BCC Research Report.

[28] Leso V., Fontana L., Mauriello M.C., Iavicoli I. (2017). Occupational risk assessment of engineered nanomaterials challenges and opportunities. Curr. Nanosci..

[29] Jiang J., Oberdorster G., Elder A., Gelein R., Mercer P., Biswas P. (2008). Does Nanoparticle Activity Depend upon Size and Crystal Phase?. Nanotoxicology.

[30] Kai W., Xiaojun X., Ximing P., Zhenqing H., Qiqing Z. (2011). Cytotoxic effects and the mechanism of three types of magnetic nanoparticles on human hepatoma BEL-7402 cells. Nanoscale Res. Lett..

[31] Baek M., Kim M.K., Cho H.J., Lee J.A., Yu J., Chung H.E., Choi S.J. (2011). Factors influencing the cytotoxicity of zinc oxide nanoparticles: Particle size and surface charge. J. Phys. Conf. Ser..

[32] Huang Y.W., Lee H.J., Tolliver L.M., Aronstam R.S. (2015). Delivery of nucleic acids and nanomaterials by cell-penetrating peptides: Opportunities and challenges. BioMed Res. Int..

[33] Chusuei C.C., Wu C.H., Mallavarapu S., Hou F.Y., Hsu C.M., Winiarz J.G., Aronstam R.S., Huang Y.W. (2013). Cytotoxicity in the age of nano: The role of fourth period transition metal oxide nanoparticle physicochemical properties. Chem.-Biol. Interact..

[34] Wu J., Sun J., Xue Y. (2010). Involvement of JNK and P53 activation in G2/M cell cycle arrest and apoptosis induced by titanium dioxide nanoparticles in neuron cells. Toxicol. Lett..

[35] Lee J.H., Ju J.E., Kim B.I., Pak P.J., Choi E.K., Lee H.S., Chung N. (2014). Rod-shaped iron oxide nanoparticles are more toxic than sphere-shaped nanoparticles to murine macrophage cells. Environ. Toxicol. Chem..

[36] Forest V., Leclerc L., Hochepie J.F., Trouvé A., Sarry G., Pourchez J. (2017). Impact of Cerium Oxide Nanoparticles Shape on their In Vitro Cellular Toxicity. Toxicol. In Vitro.

[37] Delorme M.P., Muro Y., Arai T., Banas D.A., Frame S.R., Reed K.L., Warheit D.B. (2012). Ninety-day inhalation toxicity study with a vapor grown carbon nanofiber in rats. Toxicol. Sci..

[38] Guttenberg M., Bezerra L., Neu-Baker N.M., del Pilar Sosa Idelchik M., Elder A., Oberdorster G., Brenner S.A. (2016). Biodistribution of inhaled metal oxide nanoparticles mimicking occupational exposure: A preliminary investigation using enhanced darkfield microscopy. J. Biophotonics.

[39] Oberdorster G. (2010). Safety assessment for nanotechnology and nanomedicine: Concepts of nanotoxicology. J. Intern. Med..

[40] Warheit D.B., Webb T.R., Colvin V.L., Reed K.L., Sayes C.M. (2007). Pulmonary bioassay studies with nanoscale and fine-quartz particles in rats: Toxicity is not dependent upon particle size but on surface characteristics. Toxicol. Sci..

[41] Stone V., Miller M.R., Clift M.J.D., Elder A., Mills N.L., Møller P., Schins R.P.F., Vogel U., Kreyling W.G., Alstrup Jensen K. (2017). Nanomaterials Versus Ambient Ultrafine Particles: An Opportunity to Exchange Toxicology Knowledge. Environ. Health Perspect..

[42] Nel A., Xia T., Madler L., Li N. (2006). Toxic Potential of Materials at the Nanolevel. Science.

[43] Xia T., Kovochich M., Brant J., Hotze M., Sempf J., Oberley T., Sioutas C., Yeh J.I., Wiesner M.R., Nel A.E. (2006). Comparison of the Abilities of Ambient and Manufactured Nanoparticles To Induce Cellular Toxicity According to an Oxidative Stress Paradigm. Nano Lett..

[44] Lin W., Huang Y.W., Zhou X.D., Ma Y. (2006). In vitro toxicity of silica nanoparticles in human lung cancer cells. Toxicol. Appl. Pharmacol..

[45] Lin W., Huang Y.W., Zhou X.D., Ma Y. (2006). Toxicity of cerium oxide nanoparticles in human lung cancer cells. Int. J. Toxicol..

[46] Lin W., Xu Y., Huang C.-C., Ma Y., Shannon K.B., Chen D.-R., Huang Y.-W. (2009). Toxicity of nano- and micro-sized ZnO particles in human lung epithelial cells. J. Nanopart. Res..

[47] Huang C.C., Aronstam R.S., Chen D.R., Huang Y.W. (2010). Oxidative stress, calcium homeostasis, and altered gene expression in human lung epithelial cells exposed to ZnO nanoparticles. Toxicol. In Vitro.

[48] Lin W., Stayton I., Huang Y.-W., Zhou X.-D. (2008). Cytotoxicity and cell membrane depolarization induced by aluminum oxide nanoparticles in human lung epithelial cells A549. Toxicol. Environ. Chem..

[49] Wang H.J., Growcock A.C., Tang T.H., O’Hara J., Huang Y.W., Aronstam R.S. (2010). Zinc oxide nanoparticle disruption of store-operated calcium entry in a muscarinic receptor signaling pathway. Toxicol. In Vitro.

[50] Tang T.H., Chang C.T., Wang H.J., Erickson J.D., Reichard R.A., Martin A.G., Shannon E.K., Martin A.L., Huang Y.W., Aronstam R.S. (2013). Oxidative stress disruption of receptor-mediated calcium signaling mechanisms. J. Biomed. Sci..

[51] Lai X., Wei Y., Zhao H., Chen S., Bu X., Lu F., Qu D., Yao L., Zheng J., Zhang J. (2015). The effect of Fe_2_O_3_ and ZnO nanoparticles on cytotoxicity and glucose metabolism in lung epithelial cells. J. Appl. Toxicol..

[52] Wang Y., Cui H., Zhou J., Li F., Wang J., Chen M., Liu Q. (2015). Cytotoxicity, DNA damage, and apoptosis induced by titanium dioxide nanoparticles in human non-small cell lung cancer A549 cells. Environ. Sci. Pollut. Res. Int..

[53] Periasamy V.S., Athinarayanan J., Alhazmi M., Alatiah K.A., Alshatwi A.A. (2016). Fe_3_O_4_ nanoparticle redox system modulation via cell-cycle progression and gene expression in human mesenchymal stem cells. Environ. Toxicol..

[54] Gao F., Ma N., Zhou H., Wang Q., Zhang H., Wang P., Hou H., Wen H., Li L. (2016). Zinc oxide nanoparticles-induced epigenetic change and G2/M arrest are associated with apoptosis in human epidermal keratinocytes. Int. J. Nanomed..

[55] Saptarshi S.R., Duschl A., Lopata A.L. (2013). Interaction of nanoparticles with proteins: Relation to bio-reactivity of the nanoparticle. J. Nanobiotechnol..

[56] Chang Y.-N., Zhang M., Xia L., Zhang J., Xing G. (2012). The Toxic Effects and Mechanisms of CuO and ZnO Nanoparticles. Materials.

[57] Capasso L., Camatini M., Gualtieri M. (2014). Nickel oxide nanoparticles induce inflammation and genotoxic effect in lung epithelial cells. Toxicol. Lett..

[58] Gao X., Wang Y., Peng S., Yue B., Fan C., Chen W., Li X. (2015). Comparative toxicities of bismuth oxybromide and titanium dioxide exposure on human skin keratinocyte cells. Chemosphere.

[59] Hanagata N., Zhuang F., Connolly S., Li J., Ogawa N., Xu M. (2011). Molecular Responses of Human Lung EpithelialCellstotheToxicityofCopper Oxide Nanoparticles Inferred from Whole Genome Expression Analysis. ACS Nano.

[60] Moschini E., Gualtieri G., Gallinotti D., Pezzolato E., Fascio U., Camatini M., Mantecca P. (2010). Metal oxide nanoparticles induce cytotoxic effects on human lung epithelial cells A549. Chem. Eng. Trans..

[61] Kansara K., Patel P., Shah D., Shukla R.K., Singh S., Kumar A., Dhawan A. (2015). TiO_2_ nanoparticles induce DNA double strand breaks and cell cycle arrest in human alveolar cells. Environ. Mol. Mutagen..

[62] Luo C., Li Y., Yang L., Zheng Y., Long J., Jia J., Xiao S., Liu J. (2014). Activation of Erk and p53 regulates copper oxide nanoparticle-induced cytotoxicity in keratinocytes and fibroblasts. Int. J. Nanomed..

[63] Periasamy V.S., Athinarayanan J., Alshatwi A.A. (2016). Aluminum oxide nanoparticles alter cell cycle progression through *CCND1* and *EGR1* gene expression in human mesenchymal stem cells. Biotechnol. Appl. Biochem..

[64] Ghosh M., Jana A., Sinha S., Jothiramajayam M., Nag A., Chakraborty A., Mukherjee A., Mukherjee A. (2016). Effects of ZnO nanoparticles in plants: Cytotoxicity, genotoxicity, deregulation of antioxidant defenses, and cell-cycle arrest. Mutat. Res. Genet. Toxicol. Environ. Mutagen..

[65] Patel P., Kansara K., Senapati V.A., Shanker R., Dhawan A., Kumar A. (2016). Cell cycle dependent cellular uptake of zinc oxide nanoparticles in human epidermal cells. Mutagenesis.

[66] Valdiglesias V., Costa C., Kilic G., Costa S., Pasaro E., Laffon B., Teixeira J.P. (2013). Neuronal cytotoxicity and genotoxicity induced by zinc oxide nanoparticles. Environ. Int..

[67] Xu M., Fujita D., Kajiwara S., Minowa T., Li X., Takemura T., Iwai H., Hanagata N. (2010). Contribution of physicochemical characteristics of nano-oxides to cytotoxicity. Biomaterials.

[68] Thit A., Selck H., Bjerregaard H.F. (2013). Toxicity of CuO nanoparticles and Cu ions to tight epithelial cells from *Xenopus laevis* (A6): Effects on proliferation, cell cycle progression and cell death. Toxicol. In Vitro.

[69] Setyawati M.I., Tay C.Y., Leong D.T. (2015). Mechanistic Investigation of the Biological Effects of SiO_2_, TiO_2_, and ZnO Nanoparticles on Intestinal Cells. Small.

[70] Tolliver L., Cambre M., Hou F.Y., Lee H.J., Aronstam R., Huang Y.W. (2017). Nanotoxicity of Transition Metal Oxides is a Function of Cell Killing and Suppression of Cell Proliferation. Toxicol. In Vitro.

